# Human Tick-Borne Diseases in Australia

**DOI:** 10.3389/fcimb.2019.00003

**Published:** 2019-01-28

**Authors:** Mona Dehhaghi, Hamed Kazemi Shariat Panahi, Edward C. Holmes, Bernard J. Hudson, Richard Schloeffel, Gilles J. Guillemin

**Affiliations:** ^1^Neuroinflammation Group, Faculty of Medicine and Health Sciences, Macquarie University, Sydney, NSW, Australia; ^2^Department of Microbial Biotechnology, School of Biology and Centre of Excellence in Phylogeny of Living Organisms, College of Science, University of Tehran, Tehran, Iran; ^3^Charles Perkins Centre, School of Life and Environmental Sciences and Sydney Medical School, Marie Bashir Institute for Infectious Diseases and Biosecurity, The University of Sydney, Sydney, NSW, Australia; ^4^Department of Microbiology and Infectious Disease, Royal North Shore Hospital, Sydney, NSW, Australia; ^5^Grove Health Centre, Gordon, NSW, Australia

**Keywords:** anaplasmosis, arbovirus, babesiosis, bartonellosis, Lyme-like disease, Q fever, rickettsial infection, tick paralysis

## Abstract

There are 17 human-biting ticks known in Australia. The bites of *Ixodes holocyclus, Ornithodoros capensis*, and *Ornithodoros gurneyi* can cause paralysis, inflammation, and severe local and systemic reactions in humans, respectively. Six ticks, including *Amblyomma triguttatum, Bothriocroton hydrosauri, Haemaphysalis novaeguineae, Ixodes cornuatus, Ixodes holocyclus*, and *Ixodes tasmani* may transmit *Coxiella burnetii, Rickettsia australis, Rickettsia honei*, or *Rickettsia honei* subsp. *marmionii*. These bacterial pathogens cause Q fever, Queensland tick typhus (QTT), Flinders Island spotted fever (FISF), and Australian spotted fever (ASF). It is also believed that babesiosis can be transmitted by ticks to humans in Australia. In addition, *Argas robertsi, Haemaphysalis bancrofti, Haemaphysalis longicornis, Ixodes hirsti, Rhipicephalus australis*, and *Rhipicephalus sanguineus* ticks may play active roles in transmission of other pathogens that already exist or could potentially be introduced into Australia. These pathogens include *Anaplasma* spp., *Bartonella* spp., *Burkholderia* spp., *Francisella* spp., Dera Ghazi Khan virus (DGKV), tick-borne encephalitis virus (TBEV), Lake Clarendon virus (LCV), Saumarez Reef virus (SREV), Upolu virus (UPOV), or Vinegar Hill virus (VINHV). It is important to regularly update clinicians' knowledge about tick-borne infections because these bacteria and arboviruses are pathogens of humans that may cause fatal illness. An increase in the incidence of tick-borne infections of human may be observed in the future due to changes in demography, climate change, and increase in travel and shipments and even migratory patterns of birds or other animals. Moreover, the geographical conditions of Australia are favorable for many exotic ticks, which may become endemic to Australia given an opportunity. There are some human pathogens, such as *Rickettsia conorii* and *Rickettsia rickettsii* that are not currently present in Australia, but can be transmitted by some human-biting ticks found in Australia, such as *Rhipicephalus sanguineus*, if they enter and establish in this country. Despite these threats, our knowledge of Australian ticks and tick-borne diseases is in its infancy.

## Background

Ticks and mosquitoes are recognized as the most important vectors in the transmission of bacterial and viral pathogens to humans and animals worldwide (Colwell et al., 2011). Ticks show marked genetic diversity with numerous species being mainly found in three families, *viz*. *Argasidae, Ixodidae*, and *Nuttalliellidae*. They can feed on various hosts and transmit or receive pathogenic bacteria, helminths, protozoa, and viruses to/from their host animals and humans. Although most studies have found that ticks and tick-borne illnesses are often limited to specific geographical regions, they may potentially be found anywhere in the world. International travel from endemic regions to non-endemic regions by people, animals and cargo can transport ticks. Whilst tick bites in Australia potentially can cause various diseases including bacterial and viral infections, paralysis, allergies, autoimmune disorders, post-infection fatigue and allegedly poorly quantified illnesses, the exact incidence of tick-borne disease in Australia is unknown (Graves and Stenos, 2017). Characterization of tick biology, tick-borne infections, and the distribution of ticks and tick-borne diseases can provide knowledge on their biological processes including tick immunity, reproduction, salivation, as well as tick-borne pathogens. This information is crucial for developing innovative strategies to control ticks and tick-borne disease. Understanding the microorganisms-host relationship could be exploited for our benefits (Dehhaghi et al., 2018). In case of tick-borne pathogens, such knowledge could be used for developing preventive mechanisms either for establishment of pathogens or their transmission. In this review, we will examine the geographical distribution of human-biting ticks in Australia, the reported tick-borne diseases, and potential of these ticks to carry emerging pathogens of humans and their possible transmission to humans. Allergic manifestations of tick bite are potentially life-threatening and not uncommon but are outside the scope of this paper.

## Australian Ticks

**Figure 1:**
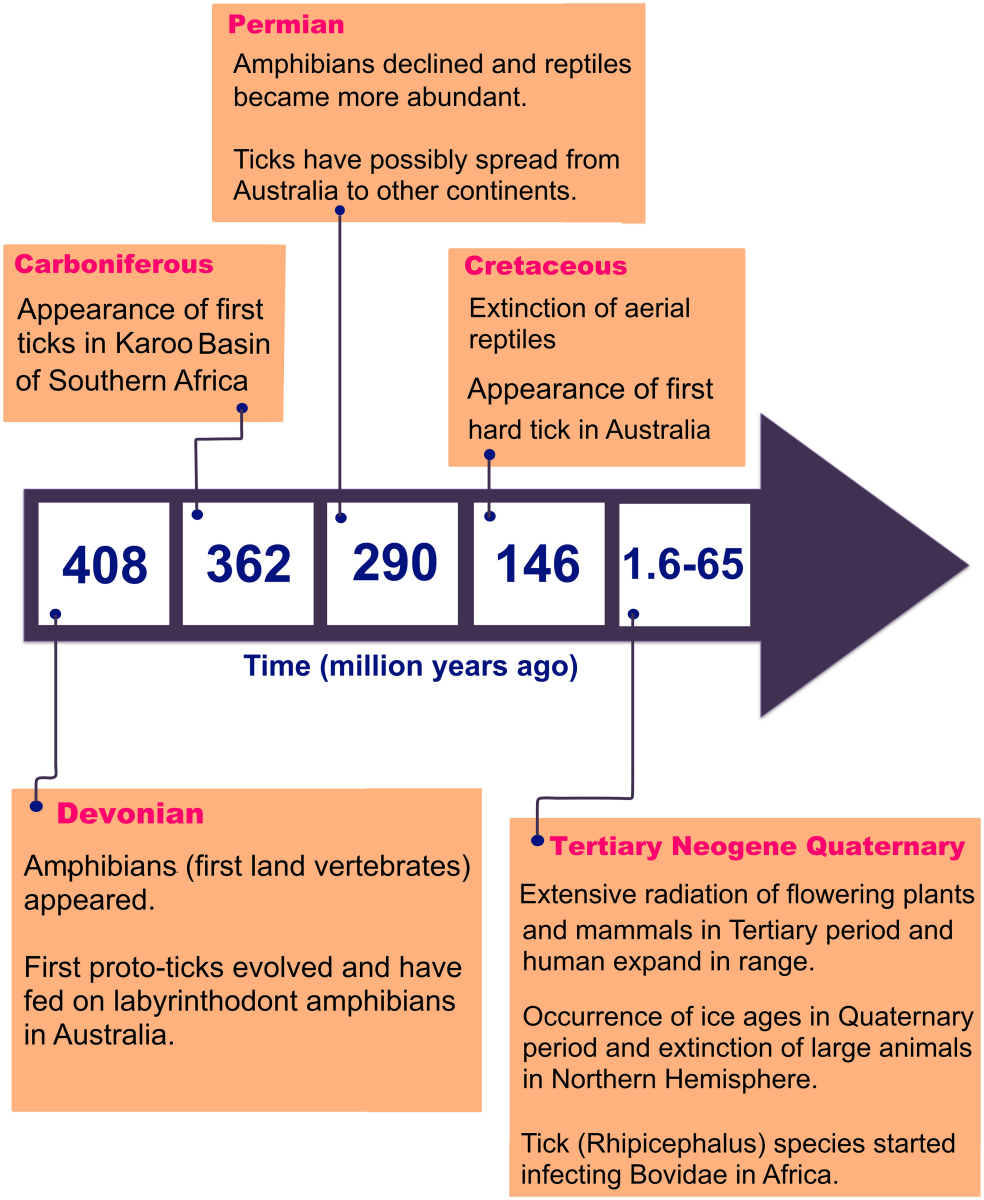
Major events in tick evolution.

There are 896 valid species of ticks worldwide, distributed in two main families of Argasidae (soft ticks) and Ixodidae (hard ticks) (Guglielmone et al., 2010; Barker et al., 2014). The major proposed events in the evolution of ticks are shown in Figure 1 (Klompen et al., 1996; Dobson and Barker, 1999; Murrell et al., 2001; Mans et al., 2012; Barker et al., 2014). Australia has unique climatic and environmental conditions that are favorable for six of the eight subfamilies of ticks including *Amblyomminae, Argasinae, Bothriocrotinae, Haemaphysalinae, Ixodinae*, and *Ornithodorinae*. Despite this faunal richness, only ~8% of all valid tick species are endemic to Australia, comprising 14 soft ticks and 58 hard ticks, mainly feeding on wildlife (Barker et al., 2014; Ash et al., 2017; Kwak et al., 2018). Of these, 17 species may attach and feed on humans and domestic animals (Table 1), whereas the remaining 55 ticks mainly feed on birds, wild reptiles, and wild mammals.

**Table 1:** Human-biting ticks of Australia with their habitats and main hosts.

**Species**	**Australian name**	**Region**	**Main host in Australia**	**References**
**Family** ***Argasidae***				
*Argas persicus*	Fowl or poultry tick	All states in Australia except Tasmania	Fowl	Roberts, 1970
*Argas robertsi*	Robert's bird tick	Lake Cowal, NSW^a^; South-western Qld^b^	Fowl, Great cormorant	Roberts, 1970
*Ornithodoros capensis*	Seabird soft tick	Along the coast from Perth, WA^c^ to Sydney, NSW; Off-shore islands, particularly coral cays of the Great Barrier Reef, Qld	Seabirds, particularly terns, gulls, penguins	Barker and Walker, 2014
*Ornithodoros gurneyi*	Kangaroo soft tick	Desert area of Australia; Malchi, Gracemere, and Brisbane, Qld	Eastern gray and red kangaroos, wallaroos	Doube, 1975
*Otobius megnini*	Spinose ear tick	WA	Domestic horses	Barker and Walker, 2014
**Family** ***Ixodidae***				
*Amblyomma triguttatum*	Ornate kangaroo tick	Northern NSW; Qld; WA; Yorke Peninsula, SA^d^	Kangaroos	Barker and Walker, 2014
*Bothriocroton auruginans*	Wombat tick	Armidale, Burrawang, and Tooloom, NSW; Benalla, Dargo (Gippsland), Healsville, Melbourne, Omeo, and Orbost, Vic^e^; Flinders Island, Deloraine, Gretna, and Tarraleah, Tasmania	Dogs, wombats	Barker and Walker, 2014
*Bothriocroton hydrosauri*	Southern reptile tick	Jenolan Caves and along the narrow state border with Vic, NSW; Eyre Peninsula and Southeastern SA; Along the coast from Bremer Bay to Albany and Margaret River area as well as along the coast from Cape Naturaliste to Cape Leeuwin, WA; Vic; Tasmania	Reptiles	Barker and Walker, 2014
*Haemaphysalis bancrofti*	Wallaby tick	Eastern-coast of Australia; Vic	Kangaroos, wallabies and their kin	Roberts, 1970; Barker and Walker, 2014
*Haemaphysalis longicornis*	Bush tick	A coastal area between Walpole and Denmark, WA; Buderim, Maleny, and Tamborine, Qld; Narrow coastal strip of eastern-coast of Australia; Taree-Wauchope region, NSW; Vic	Cattle, horses, sheep	Roberts, 1970; Barker and Walker, 2014
*Haemaphysalis novaeguineae*	-	Eastern half of Australia	Mammals	Unsworth et al., 2007
*Ixodes cornuatus*	Southern paralysis tick	Brownlee, NSW; Bullengarook, Daylesford, Donvale Warragul District, Lakes Entrance, Mallacoota, Noojee Neerim North, Orbost, Silvan, and Leongatha, Vic; Tasmania	Wide range hosts	Barker and Walker, 2014
*Ixodes hirsti*	Hirst's marsupial tick	Sub-coastal areas of southern Australia	Kangaroos and their kin, domestic dogs and cats, some birds	Barker and Walker, 2014
*Ixodes holocyclus*	Paralysis tick	Narrow coastal strip of eastern Australia; Normanton, Qld	Mammals (mainly bandicoots), Birds	Barker and Walker, 2014
*Ixodes tasmani*	Common marsupial tick	Central-eastern NSW; Qld; south-eastern SA; south-western WA; Tasmania; Vic	Australian marsupials, monotremes, rodents, domestic animals and humans	Roberts, 1970
*Rhipicephalus australis*	Australian cattle tick	Broad coastal band from north-eastern NSW to north-eastern WA	Cattle	Arundel, 1988; Barker and Walker, 2014
*Rhipicephalus sanguineus*	Brown dog tick	Most common in north of latitude 30°S; Occasionally as far as south as Sydney, NSW and Melbourne, Vic	Dogs	Roberts, 1965

a New South Wales.

b Queensland.

c Western Australia.

d South Australia.

e *Victoria*.

**Figure 2:**
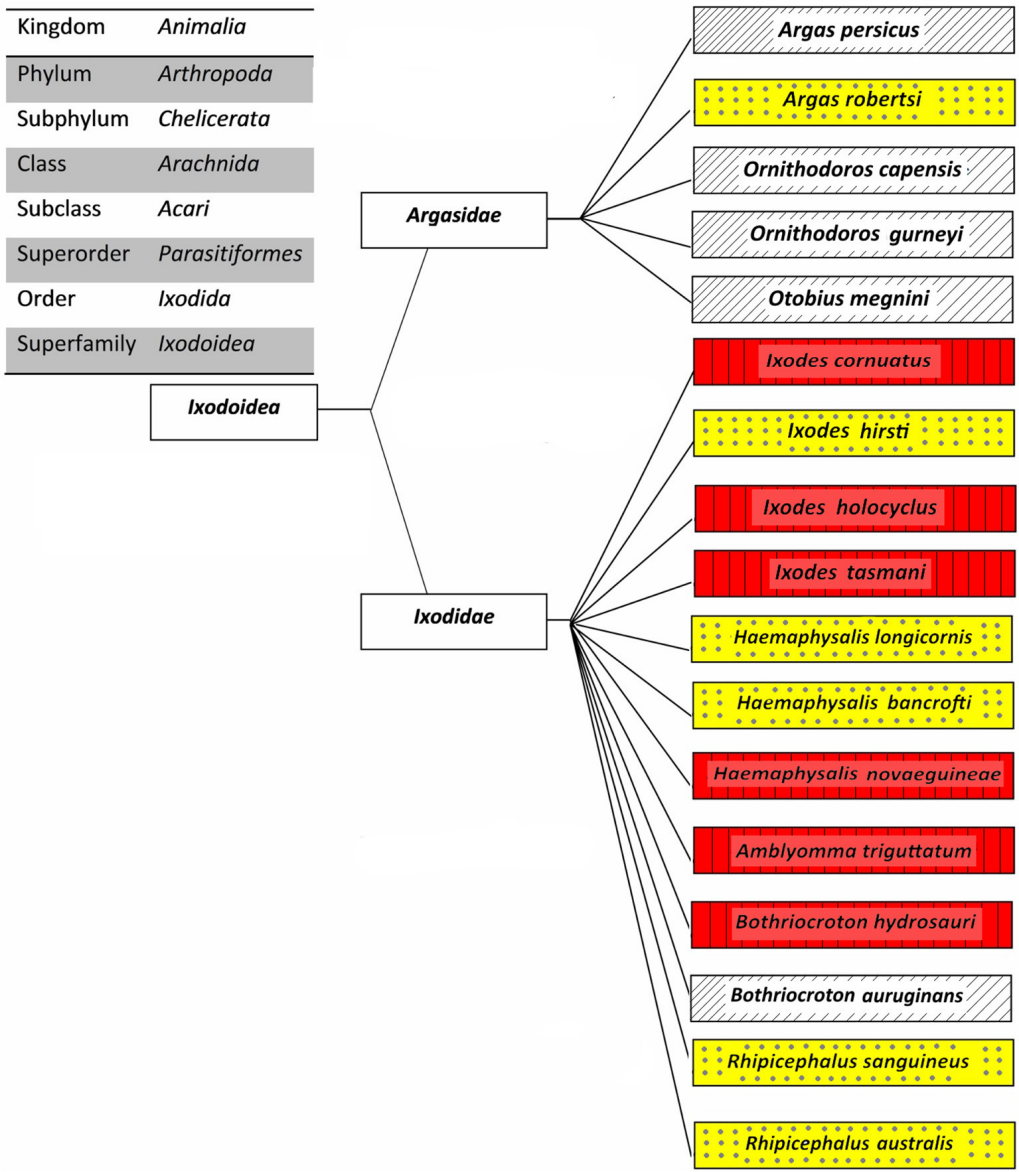
Classification of Australian human-biting ticks. Tick-borne diseases of humans that are transmitted (red boxes with vertical lines), potential tick-borne diseases of humans that may be transmitted (yellow boxes with dots), and other human-biting ticks (white boxes with upward diagonals).

The overall aim of this review is to provide relevant information on tick-borne diseases in humans; as such, only those ticks which been proven as vectors of human pathogens are discussed. The classification of 17 human-biting ticks is shown in Figure 2. Amongst them, *Argas persicus, Haemaphysalis longicornis, Otobius megnini, Rhipicephalus australis*, and *Rhipicephalus sanguineus* have been accidentally introduced into Australia by humans (Barker et al., 2014).

*Ornithodoros capensis*, previously known as *Carios capensis*, feeds primarily on seabirds, although it can bite humans if the opportunity is provided. Off-shore islands are the most likely place that this tick bites humans because they provide nesting grounds for seabirds; therefore, campers, explorers, and those who participate in recreational and professional fishing are at higher risk. *Ornithodoros gurneyi* is exclusively a desert-dwelling tick in Australia that lives mainly in the wallows of desert-dwelling kangaroos and hence rarely encounters livestock or humans. However, this tick quests in soil and ambushes humans and other mammals if they rest under a desert-tree or in a desert-cave. The bites of *O. capensis* and *O. gurneyi* cause inflammation and severe local and systemic reactions in humans, respectively. In addition, a bite from the former tick species may cause blistering, dull ache, erythema, general lassitude and discomfort, intense pruritus, lesions, lymphangitis, rheumatic pain, and weeping; whereas the latter may cause headache, impaired vision, temporary blindness, swelling, and vomiting (Henary, 1938; Barker and Walker, 2014). *O. megnini* is eyeless and may feed on people who are in close contact with horses. There are no reports of transmission of any pathogens by this tick to its hosts. However, tick spines as well as feeding in the ear canal causes considerable irritation, inflammation, and tissue necrosis of the ear which may lead to bacterial infections.

Of the 18 valid species of *Amblyomma* in Australia, only *Amblyomma triguttatum* is regularly reported on domestic animals and has been taken from humans (Barker and Walker, 2014). *Bothriocroton auruginans* is a tick with an unknown life-cycle but its larvae and nymphs may attack domestic dogs without developing any illness. However, the adult tick is strictly host specific and to date its adult form has been only found on wombats (Barker and Walker, 2014). *Bothriocroton hydrosauri*, previously known as *Aponomma hydrosauri*, is one of the most commonly studied ticks in Australia. It feeds on reptiles in southern Australia as well as cattle, horses and humans. For many years, it was believed that *H. longicornis* is a possible vector of *Theileria orientalis* in New South Wales. However, despite the reports of its ability to transmit some bacteria and viruses in other parts of the world, it is not a known vector of any pathogens in Australia or has limited vectorial capacity of *T. orientalis* (Stewart et al., 1996; Barker and Walker, 2014).

*Ixodes cornuatus, Ixodes hirsti*, and *Ixodes holocyclus* can cause paralysis in their hosts. In Tasmania, *I. cornuatus* is the only tick that has been clinically associated with paralysis and is the most common tick found on domestic animals. In contrast, *I. holocyclus* is the most common tick that causes tick paralysis in domestic animals, humans, and wildlife in Australia. Although *I. holocyclus* feeds on various birds and mammals, it needs bandicoots to sustain its life cycle and population (Barker and Walker, 2014). *Ixodes tasmani* has the most widespread geographic distribution as well as the broadest range of hosts of any Australian tick. These three species of *Ixodes* ticks occur only in Australia, with the exception of *I. cornuatus* which is also found in Papua New Guinea (Arundel, 1988; Barker and Walker, 2014). *R. australis*, previously known as *Boophilus microplus*, primarily feeds on cattle, but its larvae and young adults, especially males, may feed on humans. However, the tick is usually removed by a human host due to local irritation and itching. There is a reported case (Green, 1971) of a female *R. australis* producing viable eggs following attachment to and feeding on a human host. *R. sanguineus* is the most widespread tick in tropical and sub-tropical areas of Australia owing to its specialized feeding on domestic dogs, which are its hosts for all life stages (Barker and Walker, 2014). When dogs are not available, this tick seeks other hosts such as cattle to maintain tick populations. Additionally, the immature forms of this tick may attach to humans. This tick species can carry different human health-threatening pathogens. Some of these pathogens include *Rickettsia cornii*, the cause of “boutonneuse fever,” and *Rickettsia rickettsii*, the cause of Brazilian spotted fever and Rocky Mountain spotted fever, are not present in Australia yet.

**Figure 3:**
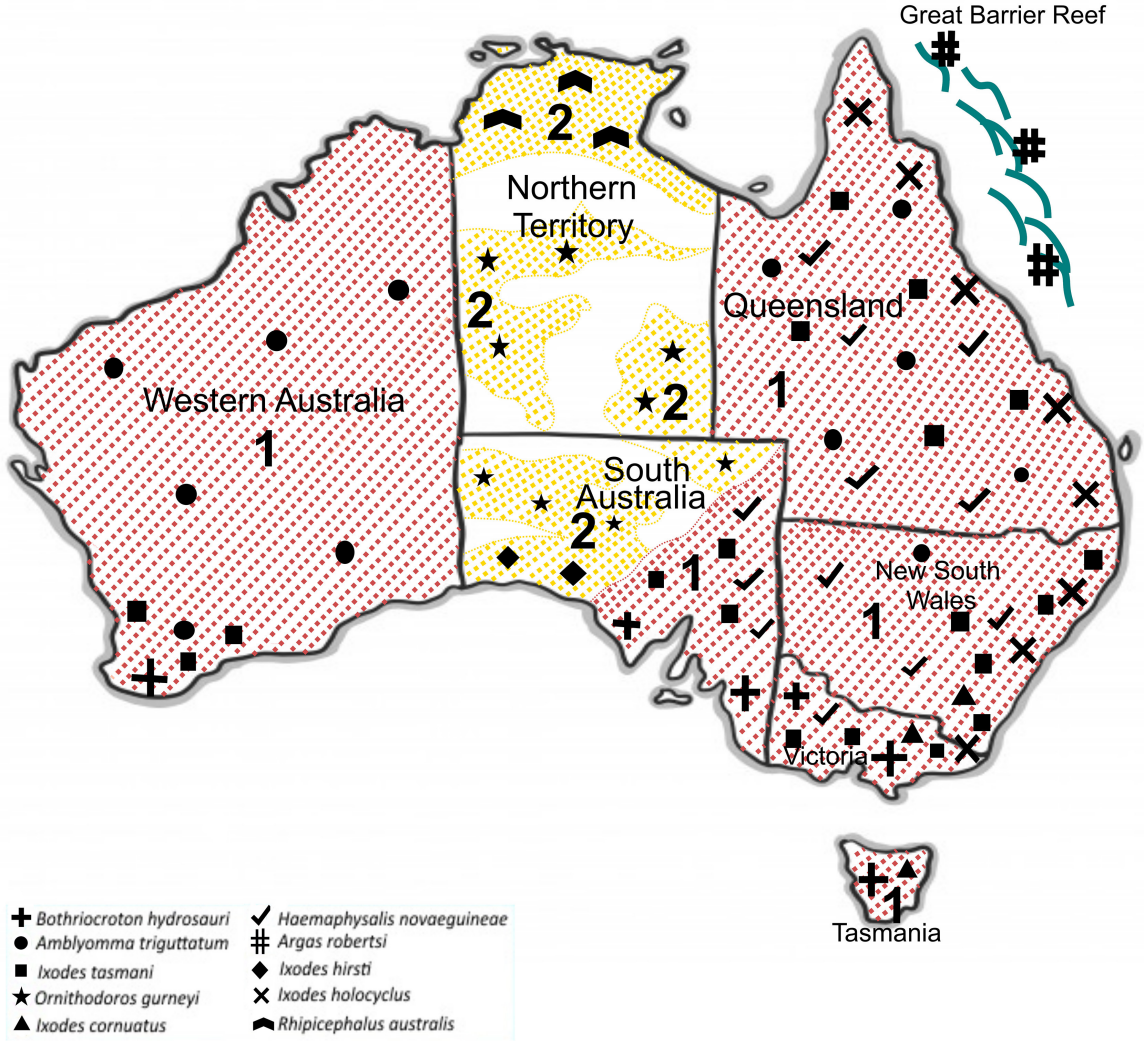
Geographical distribution of 10 potentially human biting-ticks of Australia; Tick-borne diseases of humans that are transmitted (red and 1) and potential tick-borne diseases of humans that may be transmitted (yellow and 2).

In Australia, only six out of 17 human-biting ticks act as competent vectors for the transmission of pathogens to humans. They include *A. triguttatum, B. hydrosauri, Haemaphysalis novaeguineae, I. cornuatus, I. holocyclus*, and *I. tasmani* (Barker and Walker, 2014). Figure 3 shows the geographical distribution of those six competent ticks and an additional four ticks that carry or have potential to carry human pathogens as well as the distribution of tick-borne infections of humans in Australia. It is important to note in this context that the ability to carry pathogens is different from the ability to transmit them, and active transmission has yet to be established in some cases. New South Wales, Queensland, Tasmania, Victoria, and Western Australia are endemic to at least one tick-borne infection of humans. In contrast, no tick-borne infections of humans are known to occur in north, west, and south-west portions of South Australia as well as the Northern Territory States. Significantly, the tick fauna of all states in Australia have potential to transmit new and emerging pathogens of humans. The only exceptions may be some areas within Northern Territory and South Australia States. It is unclear why no human-biting tick or tick-borne human infection has been reported from these areas. It may be because of tick density or simply lower number of field examinations. The sustainability of tick-borne pathogens within a specific geographical location is determined by tick population density, which itself is controlled by hosts population densities and tick mortality rates. The biotic (predation) and abiotic (climate including desiccation, drowning, extreme temperature) characteristics of any one location influence the host density. Moreover, environmental factors are a determinant for mortality rates of free-living tick; therefore, the suitability of specific habitat for tick population invasion, establishment, and persistence is important. For instance, larvae of *I. holocyclus* and, to lesser extent, its engorged nymphs are highly susceptible to desiccation which confines them to a narrow coastal strip with low temperatures, high humidity, and existence of hosts. It is important to emphasize that climatic patterns have direct influence on tick survival rates as mentioned earlier; critically, therefore, climate change may occasionally or permanently provide particularly favorable conditions for tick survival, increasing tick densities and exposing more humans to tick-borne pathogens. Hence, the epidemiology of ticks and tick-borne pathogens of humans also must be studied in respect to climate change and ecology. It should be also noted that any variation in fauna could change the transmission risk for tick-borne diseases through addition of new reservoir and/or amplification of the circulation of native or exotic pathogens (Marsot et al., 2013).

## Bacterial Tick-Borne Infections

**Figure 4:**
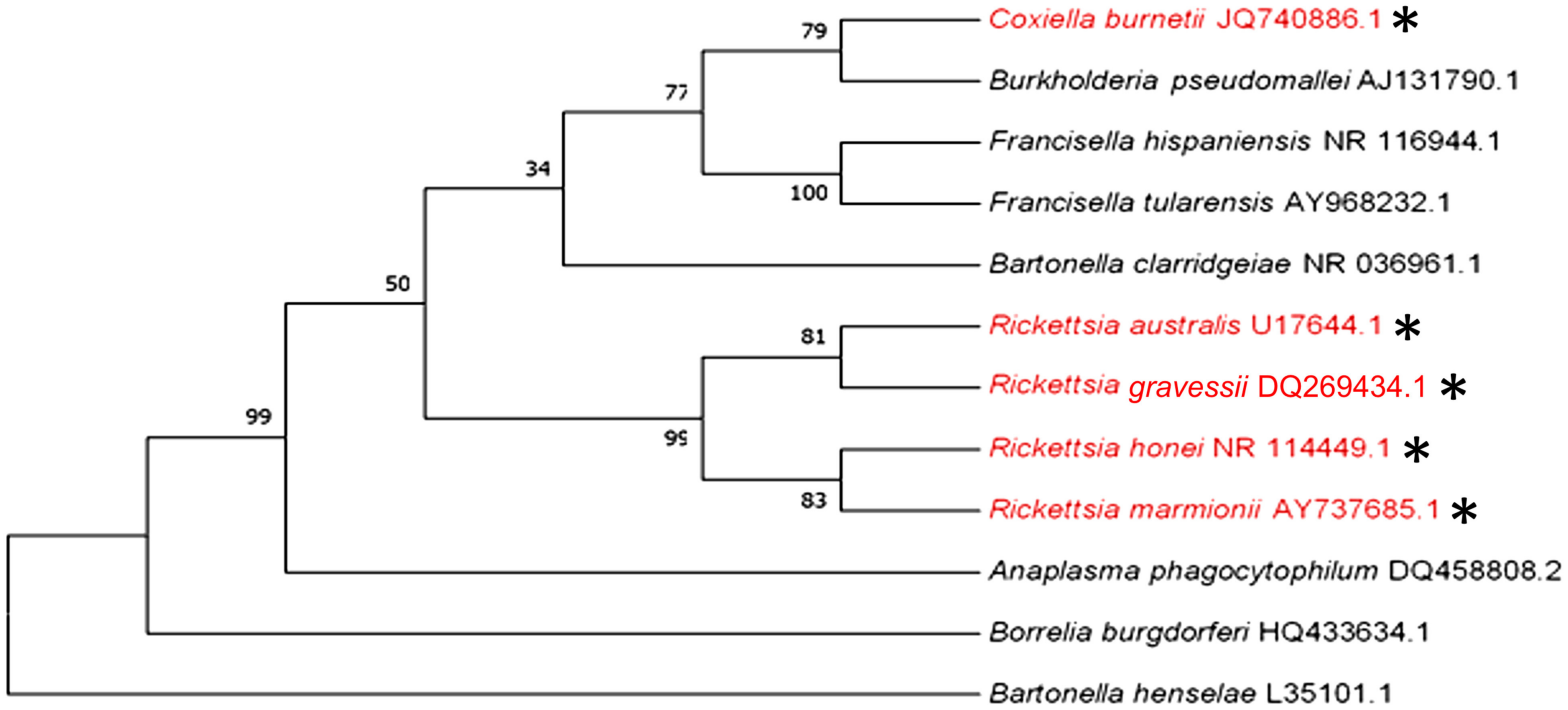
Phylogenetic analysis of pathogenic bacteria of humans that are transmitted (red and ^*^) or could potentially be transmitted by human-biting ticks (black) in Australia inferred using a Maximum Likelihood method based on the 16S rRNA gene sequence comparison (1,400 to 1,500 nucleotides).

Q fever and some rickettsial infections (see section -Q fever and Rickettsial infections) are the only bacterial diseases that are believed to be transmitted by human-biting ticks in Australia. However, ticks that bite humans may also be potential vectors for transmitting human pathogens that cause anaplasmosis, bartonellosis, Lyme-like disease, melioidosis, and tularemia in this country. The phylogenetic analysis of the causative pathogens of these diseases is shown in Figure 4.

### Q Fever

*Coxiella* is a genus of bacteria of the family Coxiellaceae, order Legionellales, class Gammaproteobacteria, and phylum Proteobacteria. *Coxiella burnetii* is the causative agent of Q fever. It was previously classified as a *Rickettsia* species due to morphological similarities. However, it has now been placed into the gamma subdivision of Proteobacteria based on genetic and physiologic characteristics, with closer similarities to *Legionella* and *Francisella* than to *Rickettsia* (Roest et al., 2013). This obligate intracellular Gram-negative bacterium protects itself in hostile environments by forming spores which can survive for long periods, for example 586 days in tick feces at room temperature (Philip, 1948). In mammals, macrophages are unable to kill *Coxiella burnetii* and the pathogen may persist asymptomatically. Furthermore, *C. burnetii* demonstrates antigenic shift, a phenomenon that is the basis of serology tests used to differentiate acute from chronic Q fever (Million et al., 2010).

Q fever notification rates decreased over 50% from 2002 to 2005, following the introduction of a nationally funded Q fever vaccination program in Australia (Gidding et al., 2009). However, this vaccine has significant side effects in persons exposed to *C. burnetii* and therefore requires pre-vaccination screening (Madariaga et al., 2003; Gidding et al., 2009). This pathogen affects a large variety of domestic (e.g., cattle, cats, goats, sheep) and wild animals as well as humans. According to Australian government Department of Health, the incidence rate of Q fever in Australia in 2005 was 17.2 per each million of the population. Currently, this disease is the most reported zoonosis in Australia. However, it should be noted that many people suffering from Q fever remain asymptomatic or only show a self-limiting febrile illness and hence are not included in calculations of incidence rate. The geographical distribution of Q fever includes Queensland and northeast New South Wales; however, it is emerging in other regions, for examples, Northern Territory and southwest Western Australia (Gidding et al., 2009).

More than 40 species of ticks can carry *C. burnetii* worldwide; there is, however, controversy over their importance in epidemiology of Q fever (Duron et al., 2015) because inhalation of infectious aerosols or dust particles remains the main route of the disease transmission. Ticks, including *Haemaphysalis bispinosa, Haemaphysalis humerosa, I. holocyclus, Rhipicephalus microplus*, and *R. sanguineus* may have roles in Q fever epidemiology in Australia (Smith, 1941). Accordingly, *H. humerosa* and *I. holocyclus* are competent vectors for *C. burnetii* and can acquire the pathogen from an infected animal and transmit it to an uninfected animal, but for the other three ticks not enough information is available to assess the vector competency for this pathogen (Smith, 1941).

The pathogen is vertically transmitted trans-stadailly from larva to nymph and from nymph to adult in the abovementioned ticks, with the exception of *H. bispinosa* and *R. microplus*. *H. bispinosa* show trans-stadial transmission only from larva to nymph (Duron et al., 2015). No information has been provided on the ability of *H. bispinosa* and *R. sanguineus* to transmit *C. burnetii* to animals. However, studies showed that *I. holocyclus* and *R. microplus* could only transmit infection from these ticks to guinea pigs by feces and bite, respectively. Despite the demonstrated transmission of *C. burnetii* in experimental systems, ticks only occasionally transmit the pathogen in the field (Duron et al., 2015). No solid information is available on tick-borne Q fever in Australian populations. Occasional case reports only suggest the possibility. For example, a human case of acute Q fever with pericarditis north-east of Perth in Western Australia has been described (Beaman and Hung, 1989) as transmitted directly by *A. triguttatum* bite. Symptoms may include abdominal and thoracic pain, bradycardia, chills, headache, high fever, myalgia, and pharyngitis after a 2–4-week incubation period. Compared to rickettsial infections, Q fever is unlikely to be associated with a rash. Apparent lung involvement may be absent as many cases present with fever, with no localizing signs, although hepatitis is common. Q fever is typically diagnosed by serology but can also be confirmed by more specialized, albeit less accessible, tests such as immunohistochemistry and polymerase chain reaction (PCR). Isolation of *C. burnetii* can only be performed in biosafety three (BSL-3) facilities, owing to its high infectivity.

### Rickettsial Infections

*Rickettsia* is a genus of non-motile, non-spore forming, obligate intracellular, Gram-negative bacteria that belongs to family Rickettsiaceae, order Rickettsiales, class Alphaproteobacteria, phylum Proteobacteria. *Rickettsia* obtains energy by parasitising vascular endothelial cells and macrophages in mammalian target organs. This pathogen can be transmitted vertically between invertebrates through life stages or be transmitted horizontally from invertebrates to vertebrates or vice versa during feeding of the tick on its host (Weinert et al., 2009). It is serologically categorized into two major classes, namely, the spotted fever group (SFG) and the typhus group. SFG rickettsia has two new sister groups: the ancestral group (AG), and the transitional group (TRG). All the members in SFG and AG, as well as *Rickettsia australis* from TRG are transmitted by tick and together these organisms encompass more than 36 tick-borne species. Of these, 15 species have been implicated as causal agents for a variety of human illnesses.

In Australia, three species including *R. australis, Rickettsia honei* (including its novel strain *Rickettsia honei marmionii*), and *Rickettsia gravesii* can be transmitted by bite of one or more ticks species, including *A. triguttatum B. hydrosauri, H. novaeguineae, I. cornuatus, I. holocyclus*, and *I. tasmani*. Unfortunately, no incidence rate has been reported for rickettsial diseases in Australia, but the annual rate of SFG rickettsioses surged up to 8.5 folds from 2008 to 2012, reaching 14.3 cases per each million populations (Drexler et al., 2016). The symptoms of these infections include eschar, fatigue, fever, headache, myalgia, and rash (macular, popular, vesicular). They are typically seen in residents of endemic areas as well as campers, travelers, and hikers to endemic areas. The severity and duration of rickettsial diseases vary considerably. Table 2 presents some information on different SFG rickettsial diseases in Australia.

**Table 2:** Spotted fever group rickettsia in Australia.

**Disease**	**Pathogen**	**Vertebrate host**	**Tick species**	**Geographical distribution**
QTT^a^	*Rickettsia australis*	Mammals (Native rats, bandicoots)	*Ixodes cornuatus* *Ixodes holocyclus* *Ixodes tasmani*	East coast of Australia with Queensland included
FISF^b^	*Rickettsia honei*	Native reptiles	*Bothriocroton hydrosauri*	Flinders Island in Tasmania; South-eastern Australia; south-western coastal of Western Australia in Salisbury Island and Walpole; south-eastern coastal region of South Australia near Adelaide
ASF^c^	*Rickettsia honei* subsp. *marmionii*	Unknown	*Haemaphysalis novaeguineae*	Eastern half of Australia
NA^d^	*Rickettsia gravesii*	Macropods and wild pigs	*Amblyomma triguttatum*	Barrow Island in north-west coast of Western Australia

a Queensland tick typhus.

b Flinders Island spotted fever.

c Australian spotted fever.

d *Not available*.

The genetic variation in Australian SFG rickettsia has been classified into two populations (Baird et al., 1996). *R. australis* and *R. honei* were designated as etiological agents of Queensland tick typhus (QTT) and Flinders Island spotted fever (FISF), respectively. Furthermore, *R. honei* strain *marmionii* causes Australian spotted fever (ASF). Whilst, ASF, FISF and QTT diseases have similar clinical and serological characteristics, their causative pathogens have varying plaque-forming abilities on different culture media. Additionally, characterization of the gene responsible for encoding the genus-specific 17-kDa antigen of *R. australis* revealed a distinct nucleotide sequence, compared to those of *R. honei* (Baird et al., 1992).

Southern blot analysis of isolates from patients with FISF and QTT showed clear differences in banding patterns when a probe for the rRNA genes is used (Baird et al., 1992). Both species respond well to antibiotic therapy with doxycycline. A new possible class of Australian SFG rickettsia has been recently proposed, following reports of possible rickettsiosis among local workers (Owen et al., 2006; Sentausa et al., 2013; Abdad et al., 2017). According to these studies, *R. gravesii* can use *A. triguttatum* as a vector to infect humans. This tick-borne disease has been reported on Barrow Island in the north-west coast of Western Australia.

Although it is also found in *Amblyomma limbatum*, no confirmed report of transmission of *R. gravesii* by this tick has been published yet. QTT is an emerging public health threat along the whole eastern seaboard of Australia. Cases may occur throughout the year. The geographical distribution of the aetiologic agent, *R. australis*, is expanding due to changes in climate and human population demographics (Stewart et al., 2017). *I. cornuatus, I. holocyclus*, and *I. tasmani* have been identified as the main vectors of this pathogen. The first description of QTT was reported from Queensland in 1946 with subsequent similar cases reported in New South Wales and Victoria (Pinn and Sowden, 1998). Generally, QTT is considered as relatively mild illness with symptoms of enlarged lymph nodes, fever, headache, maculopapular or vesicular rash, and malaise. Other possible symptoms include chills, cough, eschar, and myalgia. In 1991, a study reported the incident of SFG rickettsial infections in East Gippsland in Victoria with no identification of the causative *Rickettsia* sp. (Dwyer et al., 1991). In the same year, information on 62 Australian cases of SFG rickettsial infections from New South Wales, Queensland, and Victoria were also reviewed (Sexton et al., 1991). This included a fatal case of a healthy 68-year-old male from Mossman in Queensland (Sexton et al., 1990). The authors concluded that *R. australis* was the causative agent of all cases.

In 2007, three suspected cases of QTT were reported. Each case displayed complications including renal failure and severe pneumonia (McBride et al., 2007). More recently, five cases of QTT were reported from southern coastal New South Wales (Fergie et al., 2017), in which illness was characterized by a cutaneous eruption of erythematous papules and pustules as well as lymphadenopathy. Acute delirium or acute kidney injury was observed in three of the five cases. Improved awareness of the condition and its complications amongst the community and its clinicians is imperative to enable early diagnosis and treatment.

*R. honei* is the etiological agent of FISF (Stenos et al., 1998) and is transmitted by *B. hydrosauri*. FISF was first described on Flinders Island in Tasmania in 1991 and the causative organism was characterized (Graves et al., 1991). Symptoms include cough, fever, headache, maculopapular rash, myalgia, and transient arthralgia. FISF was initially thought to be restricted to south-eastern Australia with highest prevalence in summer, but new cases from previously non-endemic areas for this infection, including south-western coastal areas of Western Australia in Salisbury Island and Walpole, and south-eastern coastal regions of South Australia near Adelaide have been reported (Graves et al., 1991, 1993; Dyer et al., 2005; Unsworth et al., 2007).

In 2007, seven cases of SFG rickettsial diseases similar to FISF were reported from eastern Australia (Unsworth et al., 2007). Genetic identification of the etiologic agent of the disease showed close genetic relationship to *R. honei*, with also low similarities to *R. australis*. Therefore, a new strain of *Rickettsia, R. honei* subsp. *marmionii*, was designated as the causative agent of the rickettsiosis (Unsworth et al., 2007). To distinguish infection caused by *R. honei marmionii* from that of caused by *R. honei*, the name ASF was adopted. Unfortunately, no information is available on the epidemiology and ecology of its tick vector, *H. novaeguineae*, within Australia yet.

### Potential Bacterial Tick-Borne Infections

Several pathogenic bacteria have been isolated from human-biting ticks collected within Australia or have been transmitted in other parts of the globe by ticks of genera endemic in Australia. Some of these diseases, including anaplasmosis, bartonellosis, melioidosis, and tularemia have been discussed in this review. The incident rates of each of these potential disease has been provided in respect to Australia (if available) or other regions in the world to provide the readers a clue about their potential public health risks.

#### Anaplasmosis

Human granulocytic anaplasmosis (HGA), formerly known as human granulocytic ehrlichiosis, is an acute febrile disease caused by the rickettsial bacterium *Anaplasma phagocytophilum*, previously known as *Ehrlichia phagocytophilum*. This pathogen is transmitted by ticks, particularly the genera *Amblyomma, Dermacentor, Ixodes*, and *Rhipicephalus*. *A. phagocytophilum* is an obligate intracellular, Gram-negative bacterium in family *Ehrlichiaceae*, order *Rickettsiales*, class *Alphaproteobacteria*, and phylum *Proteobacteria*. This pathogen infects granulocytes and survives by suppressing or postponing vital antimicrobial mechanisms including apoptosis, oxidative burst, and phagocytosis as well as by reducing expression of defense genes in host cells. The clinical presentation is an acute, febrile, non-specific, viral-like disease with common early symptoms of headache, elevated hepatic transaminase, leukopaenia, myalgias, and thrombocytopaenia.

The incidence of HGA (cases/million/year) jumped from 1.4 in 2000 to 6.1 in 2010 and 6.3 in 2012. Although, there are as yet no reports of HGA in Australia, data is limited. Bacterial profiling of 460 ticks from four Australian human-biting tick species, namely, *A. triguttatum, Haemaphysalis bancrofti, H. longicornis*, and *I. holocyclus* were recently conducted (Gofton et al., 2015a). A novel *Anaplasma* sp. was identified in about 2% of *A. triguttatum* ticks. Other studies draw attention to the competence of *R. sanguineus* and *R. australis* in transmission of *Anaplasma* spp. (Bock et al., 1999; Rymaszewska and Grenda, 2008), both of which are also found in Australia. Further investigation is required to determine whether these ticks or other ticks within Australia can act as a vector for *A. phagocytophilum* and subsequently transmit *Anaplasma* to humans or not.

#### Bartonellosis

*Bartonella* is a genus of facultative intracellular, Gram-negative bacteria belonging to family *Bartonellaceae*, order *Rhizobiales*, class *Alphaproteobacteria*, phylum *Proteobacteria*. The three most common human diseases caused by this genus are Carrion's disease, cat scratch disease, and trench fever. The pathogenic agents of these diseases are *Bartonella bacilliformis, Bartonella henselae*, and *Bartonella quintana*, respectively. The only information about incidence of bartonellosis belongs to cat scratch disease in United States (94 cases/million people) between 2005 and 2013 (Nelson et al., 2016). These diseases are transmitted when humans are scratched by domestic or feral cats or by contact with arthropods including body louse, fleas, or sand flies. Symptoms and signs include a papule or pustule at the inoculation site, abdominal pain, bacillary angiomatosis (lesions in the skin, subcutaneous tissue, bone, or other organs), bacillary peliosis (vascular lesions in liver and spleen), bone pain, fever, enlarged lymph nodes, headache, rash, severe anemia, and subacute endocarditis. In Australia, *Bartonella clarridgeiae* and *Bartonella henselae* are found in cats, cat fleas and humans. *Bartonella henselae* sequence type 1 or strain Houston-1 is believed to be the major etiological agent of human bartonellosis and is distributed in up to 35% of the younger than 1-year cat population of Australia (Iredell et al., 2003; Arvand et al., 2007; Barrs et al., 2010; Kaewmongkol et al., 2011a). Additionally, novel *Bartonella* spp. have been identified in mammalian hosts in Australia. These include *Bartonella australis, Bartonella coopersplainsensis, Bartonella queenslandensis, Bartonella rattaustraliani, Candidatus Bartonella antechini*, isolated from eastern gray kangaroos (*Macropus giganteus*), *Uromys* spp., *Melomys* spp., *Rattus* spp., and mardo or yellow-footed antechinus (*Antechinus flavipes*), respectively (Dehio, 2008; Gundi et al., 2009; Kaewmongkol et al., 2011b).

At least eight *Bartonella* spp. are carried by some ticks within Australia, *viz. Bartonella rattaustraliani* by *Ixodes* spp., *Candidatus Bartonella antechini* n. sp. by *Ixodes antechini, Candidatus Bartonella woyliei* n. sp. by *Ixodes australiensis*, and five uncultured and unpublished *Bartonella* spp. (genotypes accession numbers EF662053 to EF662057) by perhaps *I. tasmani* (Vilcins et al., 2009; Kaewmongkol et al., 2011a). These ticks were collected from various animals, including koalas (*Phascolarctos cinereus*), rodents, woylies (*Bettongia penicillata*), or yellow-footed antechinus (*Antechinus flavipes*). Despite these findings, there is currently no convincing evidence that verifies tick-borne transmission of *Bartonella* infection to humans, in Australia. However, this possibility should not be excluded until tick-borne bartonellosis is either rejected or accepted by the performance of well-conducted, detailed studies of the relationship between humans, ticks and tick-associated *Bartonella* species (CDC, 2015).

#### Lyme and Lyme-Like Diseases

Lyme disease (or lyme borreliosis) is another tick-borne disease caused by genus *Borrelia* in family *Spirochaetaceae*, order *Spirochaetales*, and phylum *Spirochates* (Paster and Dewhirst, 2000) This spirochete is generally transmitted by *Ixodes* ticks with life-cycles that involve birds and non-human mammalian hosts (Chalada et al., 2016). The annual incidence rates of Lyme disease in England and Wales are < 2 per 100,000 (Lorenc et al., 2017), whereas that of United States is higher than 300,000 (CDC, 2017b). According to the USA CDC, this rate is based on approximately one tenth of actual cases with most remaining undiagnosed or unreported in the United States. Importantly, there is no convincing evidence for the presence of locally acquired Lyme disease in Australia. The disease can be typically initiated with an erythema migrans rash (bull's eye) at the place of tick bite followed by arthritis, influenza-like signs, and neurological disorders (Chalada et al., 2016).

The causative *Borrelia* species are classified in the *Borrelia burgdorferi* sensu lato group (Shapiro, 2014). After the initial discovery of the causative species in north-eastern USA, a number of species have been shown to cause Lyme borreliosis (Table 3). The causative bacterial species in various geographic areas are different. This highly depends on climate change in various regions that affect the number of reservoir animals, survival, and activity of ticks. In fact, the incidence of different species of *Borrelia* is determined by abundance of an appropriate vector which is dependent on climate feature of each region (Khatchikian et al., 2012). Accordingly, various species of *Borrelia* have been introduced as the main causative agent of Lyme disease in different regions. For example, *B. burgdorferi* is the only species that causes Lyme disease in the United States while in Europe and Asia other species of *Borrelia* such as *Borrelia afzelii* and *Borrelia garinii* have been also reported as Lyme disease causative agents in addition to *B. burgdorferi*. which are collectively called *B. burgdorferi* sensu lato (Shapiro, 2014).

**Table 3:** Known species from Borrelia burgdorferi sensu lato complex with potential of human Lyme borreliosis.

**Borrelia species**	**Vector**	**Geographical distribution**
*B. afzelii*	*I. ricinus, I. persulcatus*	Asia, Europe
*B. bavariensis*	*I. ricinus*	Europe
*B. bissettii*	*I. ricinus, I. scapularis, I. pacificus, I. minor*	Europe, United States
*B. burgdorferi* sensu stricto	*I. ricinus, I. scapularis, I. pacificus*	Europe, United States
*B. garinii*	*I. ricinus, I. persulcatus, I. hexagonus, I. nipponensis*	Asia, Europe
*B. kurtenbachii*	*I. scapularis*	Europe, United States
*B. lusitaniae*	*I. ricinus*	Europe, North Aferica
*B. spielmanii*	*I. ricinus*	Europe
*B. valaisiana*	*I. ricinus, I. granulatus*	Asia, Europe

**Figure 5:**
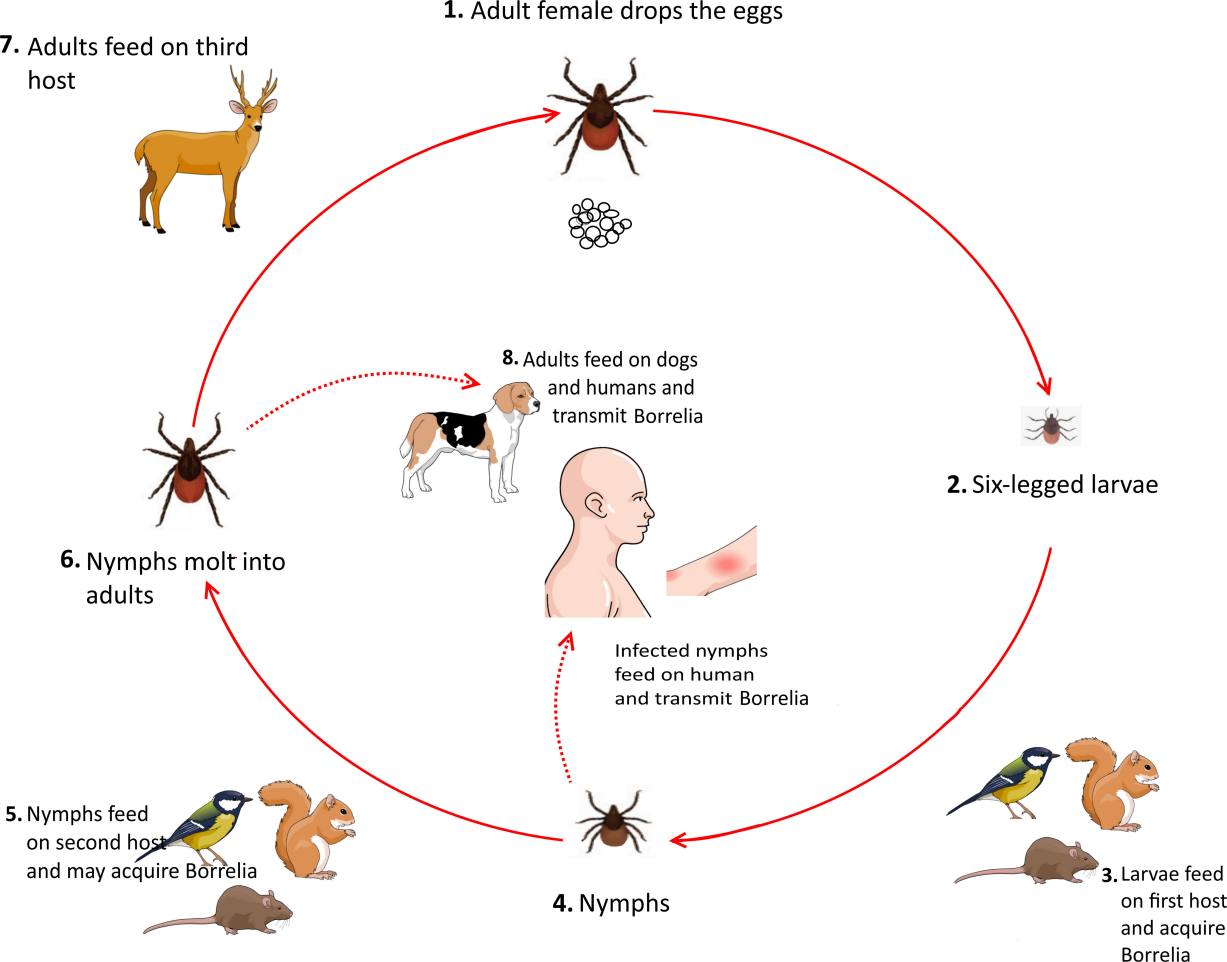
Life cycle of *Borrelia* in tick *Ixodes* spp.

Ticks usually acquire *Borrelia* by feeding on infected mice, birds, and squirrels during their larval stage. Upon the entrance to their nymphal stage, these infected ticks feed on various animals, including rodents and small mammals, which can be considered as new reservoirs for spirochaetes. After molting into the adult stage, ticks feed on larger mammals. Notably, both nymphs and adults ticks can feed on humans and cause Lyme borreliosis (Figure 5) (Donahue et al., 1987; Tilly et al., 2008).

The presence of Lyme disease (Lyme borreliosis, LB) or Lyme-like disease in Australia is highly controversial. The Australian Government Chief Medical Officer convened a Clinical Advisory Committee on Lyme Disease in 2013 to advise on aspects of Lyme disease in Australia (DOH, 2018). The first report about the presence of *Borrelia* species in Australia dates back to 1956 when a species of *Borrelia* was isolated from a rat in northern-west Queensland (Mackerras and Mackerras, 1960). *Borrelia theileri* in Queensland and New South Wales and *Borrelia anserina* in Victoria and the Northern Territory were introduced via the agricultural industry. These species are the worldwide causative agents of bovine borreliosis and avian spirochaetosis, respectively (Mulhearn, 1946).

Evidence for a vector of a potential LB pathogen in Australia is limited and there has been no research into the issue since 1994. It is assumed that if the causative species of LB is/are transmitted by ticks within Australia, likely would be (not necessarily) from the *Ixodes* genus. Research on potential vectors of LB in Australia advises that *I. holocyclus* and *I. tasmani* are the two common ticks with the widest geographical distribution in Australia (Roberts, 1970).

The presence of *Borrelia* species in ticks of Australia has been studied using various methods, including direct culture, PCR, and next generation sequencing (NGS). Wills and Barry collected 167 ticks consisting of *I. holocyclus* and *H. longicornis* from the Hunter Valley and Manning River districts of coastal New South Wales. They found rigid spirochaete-like objects (SLOs) in 41.9% of all Australian collected ticks. In addition, ELISA, immunofluorescence, and western blotting revealed that at least four bacterial isolates had similar antigenic epitopes with *B. burgdorferi*. Crucially, however, the identity of isolates was not confirmed using PCR or further sequencing (Wills and Barry, 1991).

In another Australian study, 12,000 ticks consisting of *H. bancrofti, H. longicornis*, and *I. holocyclus* were collected from the New South Wales coast and their midguts were cultured on BSK-II media. In 92 cultures straight, rigid, non-motile SLOs were detected. Further studies using electron microscopy (EM) indicated aggregates of bacterial flagellae in SLOs. Of these, 18 SLOs showed positive binding results of polyclonal *B. burgdorferi* antibodies; however, no isolates showed positive binding of monoclonal *B. burgdorferi* antibodies (Russell et al., 1994). Gofton et al. (2015b) studied 109 *I. holocyclus* ticks from around the New South Wales to find the microorganisms using PCR method. They found no member of *B. burgdorferi* sensu lato group, but their results revealed the presence of a new relapsing fever group *Borrelia* (Gofton et al., 2015b).

Hence, there is no evidence for transmission of *B. burgdorferi* sensu lato complex with Australian ticks. Whilst patients in Australia with Lyme-like disease may occasionally have positive Lyme serology, finding the causative agent using PCR or direct culture is regarded as mandatory for confirmation of local acquisition of infection.

#### Melioidosis

*Burkholderia pseudomallei* is an aerobic, non-spore forming, saprophytic motile, Gram-negative bacterium in family *Burkholderiaceae*, order *Burkholderiales*, class *Betaproteobacteria*, and order *Proteobacteria*. This bacterium is the etiological agent of melioidosis, a disease with high mortality rate (21% in Australia) because of lack of vaccine as well as significant antibiotic resistance. The resistance against antibiotics is believed to be due to secretion of highly hydrated glycocalyx capsule that contributes to formation of slime and microcolonies. This disease is endemic in northern Australia and has an incidence rate of 58/million populations during 2001-2002 (Cheng et al., 2003). The Melioidosis has 1–21 (mean 9) days incubation period (Currie et al., 2000) and its symptoms include cellulitis, fever, pneumonia, and septicemia; however, the symptoms may be absent for decades. Australian melioidosis cases have also been described in which encephalomyelitis and prostatic abscesses are not uncommon. The biogeography of *B. pseudomallei* in Australia was studied and it found two populations with sequence type diversities from Northern Territory and Queensland (McRobb et al., 2014). More than 820 documented cases of melioidosis (13% fatality) have been reported in Northern Territory over 24-year duration (Currie et al., 2010; Parameswaran et al., 2012; McRobb et al., 2014). Although, the infection is commonly transmitted through inhalation of airborne particles or inoculation, tick-borne melioidosis is not unexpected due to the susceptibilities of a broad range of animal species. *B. pseudomallei* was successfully collected from *Haemaphysalis punctate* and *Rhipicephalus bursa* after the bacterium was transmitted from infected rabbits to those ticks (Kharbov et al., 1981). Three species of *Haemaphysalis* ticks (*H. bancrofti, H. longicornis, H. novaeguineae*) and two species of *Rhipicephalus* ticks (*R. australis, R. sanguineus*) can attach to and feed on humans in Australia. Despite these facts, the role of ticks as vectors of *B. pseudomallei* has been extensively ignored in Australia. Therefore, it is crucial to validate these ticks or other Australian human-biting ticks for their ability to act as vectors for this pathogen.

#### Tularemia

Tularemia is re-emerging in many parts of world. Unfortunately, there is no report of tularemia frequency in Australian population; however, it is currently considered as an infrequent disease in the Southern Hemisphere. Among European countries, the highest incidence rate of 52/million belonged to Kozovo during 2001–2010. It was followed by (per million people) Sweden, 28; Finland, 11.9; Slovakia, 10.0; Czech Republic, 8.1; Norway, 4.2; Serbia-Montenegro, 4.0; Hungry, 3.6; Bulgaria, 2.1; and Croatia, 1.5. The rate for United States is 0.5–5 for same number of population (Gürcan, 2014).

Tularemia is caused by bacterium *Francisella tularensis* or its subspecies. The genus *Francisella* is a member of family *Francisellaceae* in order *Thiotrichales*, class *Gammaproteobacteria*, and phylum *Proteobacteria*. There are four subspecies of this aerobic, facultative, intracellular, non-motile, non-spore forming, Gram-negative bacterium, namely, *F. tularensis tularensis* (type A), *F. tularensis holarctica* (type B), *F. tularensis mediasiatica*, and *F. tularensis novicida*. Where present, it is a highly contagious pathogen in domestic animals and humans. It persists in soil and water and ubiquitously occurs in arthropod vectors as well as wildlife. Tularemia may be transmitted through direct contact, ingestion or inhalation, and indirectly through bites of infected deer flies, ticks, or even infected animal. The disease is categorized into six groups of glandular, oculoglandular, oropharyngeal, pneumonic, typhoidal, and ulceroglandular (CDC, 2017a). Symptoms may appear 3–5 days after exposure and include abdominal pain, anorexia, chest discomfort, cough, chills, diarrhea, fatigue, fever, headache, malaise, myalgia, sore throat, and vomiting. The bite of an infected tick or animal causes an ulceroglandular form of tularemia, in which pain and inflammation develops at the bite site accompanied by enlargement of nearby lymph nodes. In Australia, the ringtail possum (*Pseudocheirus peregrinus*) is, so far proven to be, the only natural host of tularemia.

In 2011, a case of infection with *F. tularensis holarctica* that was transmitted through bites of an infected ringtail possum in Tasmania was reported (Jackson et al., 2012). Six years later, *F. tularensis holarctica* was isolated from ringtail possums in Sydney (Eden et al., 2017). Additionally, two *Francisella* spp. were separately isolated from two infected women in Australia and both isolates were identified as *Francisella hispaniensis* (Whipp et al., 2003; Aravena Román et al., 2015). These studies proved the presence of *Francisella* spp. in New South Wales, Northern Territory, Tasmania, and Western Australia at least since 2003. The known tick vectors for tularemia are *Amblyomma americanum, Dermacentor andersoni*, and *Dermacentor variabilis*. Although these ticks are not present in Australia, ringtail possums are hosts for some ticks that bite humans e.g., *I. hirsti* and *I. holocyclus*. Therefore, researchers must thoroughly evaluate these ticks for their ability to acquire endemic *Francisella* spp. from ringtail possums and then transmit them to humans. Additionally, it will be important to evaluate whether additional animals, such as kangaroos or domestic animals, may act as possible hosts for *Francisella* sp. as well as the possibility of indirect transmission to humans by ticks which in Australia that have both humans and these animals as hosts.

## Viral Tick-Borne Infections

Arboviruses (arthropod-borne viruses) are a major public health concern in Australia, with more than 75 arboviruses identified in Australia, some of which are associated with human diseases, and are almost exclusively female mosquito-borne (Russell and Dwyer, 2000; Smith et al., 2011). Dera Ghazi Khan virus (DGKV), *I. holocyclus* Iflavirus (IhIV), Lake Clarendon virus (LCV), Saumarez Reef virus (SREV), Upolu virus (UPOV), and Vinegar Hill virus (VINHV) are, to date, the only reported viruses endemic in Australia that have been isolated from human-biting ticks. DGKV was isolated from *Argas robertsi* in Darwin (Northern Territory), whereas LCV and VINHV were independently taken from the same tick species that were attached to and fed on the eastern subspecies cattle egrets (*Bubulcus ibis coromandus*) and the western cattle egrets (*Bubulcus ibis*) at Gatton in south-east Queensland, respectively (Doherty et al., 1976; St George et al., 1984; Gauci et al., 2017).

LCV belongs to genus *Orbivirus*, family Reoviridae (double-stranded RNA virus). DGKV and VINHV consist of three negative-sense, single-stranded RNA that classified in genus *Orthonairovirus*, family Nairoviridae, and order Bunyavirales. It is worthy of mention that the genus *Orthonairovirus* includes several viruses that are associated with severe infections in humans or other vertebrate hosts like Crimean-Congo haemorrhagic fever and Nairobi sheep disease. SREV and UPOV were independently isolated from *O. capensis* associated with sooty terns (*Onychoprion fuscatus*) on coral cays off the east coast of Queensland and on Upolu Cay in a coral atoll of Great Barrier Reef in Queensland, respectively (George et al., 1977).

SREV was also isolated from *Ixodes eudyptidis* associated with a dead silver gull (*Chroicocephalus novaehollandiae*) in Northern Tasmania. However, as *I. eudyptidis* does not attach to and feed on humans, it is not considered further in this review. SREV belongs to genus *Flavivirus* in the family *Flaviviridae* that possesses positive-sense single-stranded RNA. The genus *Flavivirus* includes arboviruses such as dengue virus, tick-borne encephalitis virus (TBEV), yellow fever virus, Zika virus, and West Nile virus that can cause severe illnesses in humans.

Tick-borne encephalitis (TBE) is caused by TBEV that is transmitted by consumption of unpasteurized/raw milk as well as bites of *Ixodes* ticks, although there is no evidence that it exists in Australia aside from those who have been infected overseas. TBE is a systemic disease of humans, with a pronounced effect on the central nervous system, and complications are not unusual (Brown, 1994). The virus may access the central nervous system by either haematogenous or neuronal routes. Since its first isolation in 1937, three virus sub-types have been described; namely, European or Western TBEV, Far eastern TBEV (previously known as Russian Spring Summer encephalitis virus), and Siberian TBEV (CDC, 2014). TBE was characterized in an Australian man following a 6-week trip traveling through Russia (Chaudhuri and Růžek, 2013).

*Iflaviridae* is another family that belongs to Group IV (positive-sense, single-stranded RNA) viruses with *Iflavirus* as the sole genus member. Recently, a novel member of this genus, i.e., IhIV has been identified from *I. holocyclus* populations from northern New South Wales and Queensland. Currently, no human disease has been caused by any members of family *Iflaviridae* (O'Brien et al., 2018). Almost 50 years after isolation of UPOV, an enveloped spherical virus, (Briese et al., 2014) provided clear demonstration that UPOV is a member of *Thogotovirus* in family *Orthomyxoviridae*. Its genome consists of six segments of negative-sense, single-stranded RNA (Group V). UPOV extensively infects African green monkey kidney, baby hamster kidney, human embryonic kidney 293 and is lethal to newborn mice when inoculated intracerebrally (Doherty et al., 1969; Briese et al., 2014). Despite the apparent lack of pathogenicity factor, UPOV can cause disease in humans. Furthermore, a novel *Thogotovirus* member, Bourbon virus, was most likely transmitted by tick bite to a healthy man on North America and took his life within 11 days under medical care due to cardiopulmonary arrest (Kosoy et al., 2015). He was unresponsive to doxycycline therapy and showed fatigue, fever, multiorgan failure, leukopenia, and thrombocytopenia.

Despite the isolation of all the aforementioned arboviruses more than 35 years ago, no information on their pathogenicity for humans is available. RNA viruses are abundant infectious agents that can be transmitted by about 10% of all species of tick in the world, owning to highly specific nature of the relationships among viruses, ticks, and vertebrate hosts. RNA viruses result in more fatalities than tick-borne microbes. Therefore, comprehensive surveillances and characterizations of these viruses to carefully monitor their potential as emerging pathogens in regard to virus survival and its ability to replicate and infect both tick and human cells are crucial. Metagenomic sequencing technology now offers a way of effectively screening samples for the presence of potential tick-borne human viruses. The potentially serious health consequences of infection with these viruses highlights the vital need for comprehensive surveillance for these viruses and any potential clinical illness caused by them, as more investigation is required to determine their potential to be emerging pathogens.

## Other Tick-Borne Diseases

### Babesiosis

Among more than 100 *Babesia* spp. reported worldwide, only a few species including *B. divergens, B. duncani, B. microti*, and *B*. MO-1 can cause disease in humans. Of these, *B. divergens* and *B. microti* have been identified in most human babesiosis. In Australia, *B. duncani* and *B. microti* have been identified through sequencing and/or serology (Sanjaya et al., 2012). The genus *Babesia* is classified in family *Babesiidae*, order *Piroplasmida*, class *Aconoidasida*, phylum *Apicomplexa*. This protozoan is transmitted by ticks, mainly *Ixodes scapularis* (not present in Australia), and is the most common cause of babesiosis in humans. It can either reproduce asexually in its mammalian host erythrocytes or sexually in its arthropod vector. No information on its incidence rate in Australia or in world is available; however, its incidence rate is lower than that of Lyme diseases due to higher difficulty in diagnosis, higher proportion of asymptomatic infection, inadequate physician awareness, lower tick infection rate, and more restricted geographic range (Vannier et al., 2015). This protozoan is the parasite of human red blood cells. Within these cells, trophozoites of *B. microti* reproduce by budding and undergo two successive divisions to form Maltese Cross (tetrad morphology). Then, merozoites are release into bloodstream, with simultaneous lysis of red blood cells, and attach to and invade other red blood cells. Although babesiosis is a well-documented infection of domestic animals including cattle and dogs, there is, to date, only one human case has been reported from Australia. The case was identified on the south coast of New South Wales in 2012 (Sanjaya et al., 2012). This patient developed cholestatic liver function disorder, moderate-to-severe thrombocytopenia, pancytopenia with fluctuating anemia, multiorgan failure, and required ongoing blood product transfusions as well as haemodialysis. The patient did not recover from multiorgan failure, and severe thrombocytopenia led to acute gastrointestinal bleeding and a fatal asystolic arrest (Sanjaya et al., 2012). The causative agent was confirmed as *B. microti* through complete and partial sequencing of the 18S rRNA gene (18S rDNA) and the β-tubulin gene. Asymptomatic parasitaemia is common in babesiosis during primary infection and/or following treatment of systemic infection (Vannier et al., 2008; Sanjaya et al., 2012). This disease is primarily transmitted through the infected tick bites (commonly *Ixodes* spp.) with occasional transmission through the transfusion of blood products. Unlike several countries such as US, blood products are not screened for *Babesia* in Australia, which pose the danger of transmission through blood donation by unrecognized babesiosis patients (Ngo and Civen, 2009; Government, 2018). Patients may have fever, haemolytic anemia, influenza-like disease, and thrombocytopenia. Asplenics typically are susceptible to severe life-threatening illness.

Babesiosis may be suspected in Australia in patients with a history of overseas travel to an endemic area, with or without a documented history of tick bite, or a history of blood transfusion. A tick-borne route was assumed because the parasitaemia was neither transfusion related nor acquired overseas (Sanjaya et al., 2012). A later study (Storey Lewis et al., 2017) extracted DNA from 1,154 ticks that where collected from across Australia to characterize *Babesia* spp. However, no *B. microti* could be identified in these Australian ticks. Only several closely related sequences to *B. macropus* were reported from ticks of genera *Bothriocroton, Haemaphysalis*, and *Ixodes*. Therefore, the animal host and tick vector are yet to be identified in Australia.

### Tick Paralysis

Tick paralysis is the only tick-borne disease that is not attributed to pathogens. The bite of a single tick is sufficient to paralyze one animal. The injection of chemical compounds, i.e., neurotoxins and paralysis is usually due to the attachment of an adult female *I. holocyclus*, mostly in the spring and summer months. Larva of *I. holocyclus* cannot feed on humans; however, their attachments to humans usually cause no more than localized dermatitis. For example, a larva of *I. holocyclus* was found attached to right temporal conjunctiva of a 10-year-old boy from Sydney in New South Wales (Teong et al., 2015). The only symptoms developed were eye itch and conjunctival vessel dilatation around the organism. In contrast to larva, nymphs and to a greater extent female *I. holocyclus*, frequently attach to humans and after several days can abundantly feed and engorge (Barker and Walker, 2014). During a blood meal, especially after day three of feeding, sufficient chemical compounds including neurotoxins (holocyclotoxin), secreted by female tick salivary glands, are injected into hosts. The neurotoxin may bind to the location where nerves meet muscles, i.e., neuromuscular junction and reduces the release of acetylcholine at the presynaptic membrane which may lead to acute anaphylactic shock and paralysis (Chand et al., 2016).

Tick paralysis extensively occurs in Australia and many researchers have reported human case studies. It is worth mentioning that the geographical distribution of such cases is highly restricted to the enzootic range of the paralysis tick. The most commonly affected group is children 1–5 years of age and infected children usually become subdued, refuse food, and sleep excessive periods (Grattan Smith et al., 1997). The typical presentation is a prodrome with the subsequent development of an unsteady gait followed by ascending, symmetrical, flaccid paralysis (Grattan Smith et al., 1997). The disease is characterized by early cranial nerve involvement, especially, with the presence of both internal and external ophthalmoplegia (Grattan Smith et al., 1997). It is noteworthy that the progress of paralysis commonly continues for 24–48 h after removal of Australian paralysis tick, unlike the short duration seen with North American ticks. Therefore, it is crucial to carefully observe the affected child during this period. Since the second-half of the twentieth century, death due to respiratory failure has been relatively uncommon if appropriate early diagnosis and supportive medical care are provided.

No documented fatality has been reported in Australia since 1945. Respiratory support may be needed for more than a week (Grattan Smith et al., 1997) and a recovering child requires several weeks to walk unaided. It should be pointed out that currently no study has ever been investigated tick paralysis recovery in a longitudinal pattern. In older children and adults, the initial symptoms may be difficulty in reading with double vision, nystagmus, or photophobia (Sutherland and Tibballs, 2001; Barker and Walker, 2014). According to these reports, there is no increase in body temperature unless the disease is complicated by bacterial infection.

There have been three exported cases of *I. holocyclus* attachments reported in the literature, although there are probably other cases that have occurred in the past but were not recognized. The first case was a Japanese man traveled to the Gold Coast in Queensland in late 2002 (Inokuma et al., 2003). After his return to Japan, he removed a semi-engorged female tick that had attached to his scalp 3 days earlier. The patient developed an illness consistent with SFG rickettsia, but without rash. Serology was positive for antibodies to SFG, but PCR did not detect rickettsia in the tick. The second case was reported in 2014 (Pietzsch et al., 2014) from an English traveler returning back to London from East coast of Australia. She displayed swelling and a small black lump in the groin area. The ticks were finally discovered and removed from her lower leg. Two years later, a paralysis tick was attached to a woman's right temporal region of scalp following a trip to Sydney (Pek et al., 2016). She presented to a Singapore Emergency Department (ED) with facial swelling, facial pain and a painful, swollen skin tag over her right temporal region, that proved to be a female adult *I. holocyclus* tick. She developed motor and sensory changes with weakness in the distribution of the temporal branch of the facial nerve with dysesthesiae over scalp in the sensory distribution of that nerve (Pek et al., 2016).

## Conclusions

The Australian climatic and environmental conditions provide suitable conditions for many ticks. Australia is known to be endemic for 70 of the known 896 tick species. At least 17 species of Australian ticks attach to and feed on domestic animals and humans. Although humans are accidental hosts for many of these ticks, tick bites may have negative influences on human health and quality of life. Whilst tick-borne pathogens of humans do not appear to contribute to Australia's overall communicable disease burden, this perception must be re-examined using new laboratory and epidemiological tools that we now have at our disposal. Against a true baseline estimate of the burden of illness associated with tick bite, we can then prepare for the future when changes in climate, lifestyle, human and animal populations will invariably impact on the likelihood that tick bite will likely increase in many parts of Australia. Introduced species of ticks, many of which harbor pathogens not previously seen in Australia, may adapt to and flourish in Australia. *A. persicus, H. longicornis, O. megnini, R. australis*, and *R. sanguineus* are good examples of species introduced into Australia because of human interventions. Human-biting ticks carry pathogens such as arboviruses (DGKV, LCV, SREV, TBEV, UPOV, VINHV) as well as *Anaplasma, Borrelia, Burkholderia, Francisella*, and *Rickettsia* species. These pathogens have been identified in some human-biting ticks such as *A. robertsi, H. bancrofti, H. longicornis, I. hirsti, R. australis*, and *R. sanguineus* that have been collected within Australia. Some of these introduced human-biting ticks that are now endemic to Australia can carry serious human pathogens that have not yet been detected in Australia but are well-known in other parts of the globe. *R. sanguineus* is a vector of *R. conorii* and *R. rickettsii*, both causes of SFG infections that have higher fatality rates than ASF. New pathogens may be introduced into and then established in Australia by many means that are not amenable to simple regulation, such as tourism, trade, bird or animal migration.

Aside from pathogen transmission, there are an increasing number of allergic, inflammatory and potentially autoimmune illnesses attributed to tick bites. *I. holocyclus, O. capensis*, and *O. gurneyi* are three tick species that trigger such complications in humans and are also present in Australia. Currently, only some areas in Northern Territory and South Australia may be free from human-biting ticks and tick-borne diseases. Tick-borne infections and illness have been reported in all other states including New South Wales, Queensland, Tasmania, Victoria, and Western Australia. Accordingly, Australia undoubtedly faces new disease threats associated with tick-bite, all of which can only be countered by improving our knowledge of the ticks, the pathogens and the epidemiology of tick bite and its consequences. There is an urgent need for a comprehensive study to determine the role of Australian human-biting ticks in the transmission of emerging pathogens to humans in Australia.

## Author Contributions

MD and HK wrote the manuscript and prepared the figures. EH, RS, BH, and GG have reviewed and corrected the manuscripts based on their respective expertise. All authors read and approved the final version of the manuscript.

### Conflict of Interest Statement

The authors declare that the research was conducted in the absence of any commercial or financial relationships that could be construed as a potential conflict of interest.

## Abbreviations

AG: Ancestral Group
ASF: Australian Spotted Fever
BSK-II: Barbour Stoenner Kelly II
DGKV: Dera Ghazi Khan Virus
FISF: Flinders Island Spotted Fever
HGA: Human Granulocytic Anaplasmosis
LCV: Lake Clarendon Virus
QTT: Queensland Tick Typhus
SREV: Saumarez Reef Virus
SLO: Spirochaete-Like Object
SFG: Spotted Fever Group
TBE: Tick-Borne Encephalitis
TBEV: Tick-Borne Encephalitis Virus
TRG: Transitional Group
UPOV: Upolu Virus
VINHV: Vinegar Hill Virus.
